# BRG1 interacts with GLI2 and binds *Mef2c* gene in a hedgehog signalling dependent manner during in vitro cardiomyogenesis

**DOI:** 10.1186/s12861-016-0127-8

**Published:** 2016-08-02

**Authors:** Joel Vincent Fair, Anastassia Voronova, Neven Bosiljcic, Rashida Rajgara, Alexandre Blais, Ilona Sylvia Skerjanc

**Affiliations:** 1 Department of Biochemistry, Microbiology and Immunology, Faculty of Medicine, University of Ottawa, 451 Smyth Rd, K1H 8M5 Ottawa, Canada; 2 Ottawa Institute of Systems Biology, University of Ottawa, 451 Smyth Rd, K1H 8M5 Ottawa, Canada

**Keywords:** Cardiac development, Cardiomyogenesis, Hedgehog signalling pathway, GLI2, BRG1

## Abstract

**Background:**

The Hedgehog (HH) signalling pathway regulates cardiomyogenesis in vivo and in differentiating P19 embryonal carcinoma (EC) cells, a mouse embryonic stem (mES) cell model. To further assess the transcriptional role of HH signalling during cardiomyogenesis in stem cells, we studied the effects of overexpressing GLI2, a primary transducer of the HH signalling pathway, in mES cells.

**Results:**

Stable GLI2 overexpression resulted in an enhancement of cardiac progenitor-enriched genes, *Mef2c*, *Nkx2-5*, and *Tbx5* during mES cell differentiation. In contrast, pharmacological blockade of the HH pathway in mES cells resulted in lower expression of these genes. Mass spectrometric analysis identified the chromatin remodelling factor BRG1 as a protein which co-immunoprecipitates with GLI2 in differentiating mES cells. We then determined that BRG1 is recruited to a GLI2-specific *Mef2c* gene element in a HH signalling-dependent manner during cardiomyogenesis in P19 EC cells, a mES cell model.

**Conclusions:**

Thus, we propose a mechanism where HH/GLI2 regulates the expression of *Mef2c* by recruiting BRG1 to the *Mef2c* gene, most probably via chromatin remodelling, to ultimately regulate in vitro cardiomyogenesis.

**Electronic supplementary material:**

The online version of this article (doi:10.1186/s12861-016-0127-8) contains supplementary material, which is available to authorized users.

## Background

The embryonic heart is the first organ to develop and is essential for life. Perturbations in heart development can lead to congenital heart disease, which is the most common birth defect in the Western world [1]. During heart development, cardiac precursors populate the first and the second heart fields (FHF and SHF), which establish the cardiac crescent by embryonic day (E) 7.5 [2, 3]. In the cardiac crescent a transcriptional network regulates cardiac progenitor differentiation in a spatiotemporal manner through a crosstalk of inductive signals from surrounding tissues, including hedgehog (HH) [4, 5]. These signals lead to the expression of cardiac progenitor-enriched transcription factors, including NK2 homeobox 5 protein (NKX2-5), myocyte-specific enhancer factor 2C (MEF2C), T-box protein 5 (TBX5), and GATA-binding protein 4 (GATA-4), which are essential for efficient heart looping and morphogenesis [5–10].

When one of the three mammalian HH ligands - Indian (IHH), desert (DHH), or sonic hedgehog (SHH) - binds to and inhibits the transmembrane protein, patched 1 (PTCH1) [11, 12], smoothened (SMO) initiates the transition of glioma-associated (GLI) proteins into the nucleus for modulation of HH target gene expression [13]. GLI2 is a primary transducer and activator of the response to HH signalling [13]. GLI1 is a transcriptional activator but its expression is dependent on GLI2 and/or GLI3 [13]. In the absence of HH ligand, PTCH1 inhibits SMO activity, which triggers phosphorylation of the GLI proteins and results in their proteolytic cleavage and/or degradation [14]. While phosphorylation of GLI3 mostly yields a partially cleaved GLI3 transcriptional repressor (GLI3R), which represses the expression of HH target genes, the phosphorylation of GLI2 mainly leads to its complete proteasomal degradation [13].

*Shh*^*-/-*^ mice have altered heart looping [15] and a single outflow tract [16]. Mice with tissue specific knockout of *Shh*^-/-^ in the SHF, using *Nkx2-5-Cre*, display outflow tract septation defects [17]. *Shh*^*-/-*^*/Ihh*^*-/*-^ or *Smo*^*-/-*^ embryos have a delayed *Nkx2-5* expression and heart tube formation [18]. In accordance, *Ptch1*^-/-^ embryos, which have the negative regulation of HH signalling removed, express higher levels of *Nkx2-5* in the cardiac crescent [18]. *Gli2*^-/-^*Gli3*^+/-^ embryos have persistent truncus arteriosus (PTA) and a single outflow tract [19, 20]. SMO agonist (SAG), which enhances HH signalling, increases embryonic chick cardiac progenitor cell proliferation in vivo and in vitro [21]. In zebrafish, treatment with SAG or *Shh* enhances the number of cardiomyocytes in the developing cardiac chambers [22, 23], whereas treatment with the HH signalling inhibitor, cyclopamine, reduces *Nkx2-5* and *Myhc* expression as well as cardiomyocyte proliferation [22, 23]. Together these studies demonstrate that functional HH signalling is important for regulating the number of cardiac progenitor cells and heart development in vivo.

*D. melanogaster* embryos lacking a single *Mef2* gene do not exhibit any muscle development [24]. In mammals, there are four MEF2 members, MEF2A-D [25]. Expression of a dominant-negative fusion protein of MEF2C with an engrailed repression domain (EnR) under the regulation of an *Nkx2-5* enhancer (*Nkx2-5*-MEF2C/EnR), which mediates the repression of all MEF2 target genes, leads to severely disrupted cardiomyogenesis in mice [26]. *Mef2c*^-/-^ mice or mice with myocardium-specific knockout of *Mef2c,* through either *Myhc6-Cre* or *Mlc2v-Cre,* fail to undergo heart looping morphogenesis, as well as correct development of the right ventricle and outflow tract [8, 9]. Thus, MEF2 factors are important for early heart development.

Differentiating mouse embryonic stem (mES) cells share a similar hierarchical set of gene expression patterns observed during cardiomyogenesis in vivo [27]. The mesoderm marker, *Brachyury*, and the precardiac mesoderm marker, *Mesp1,* are expressed by days 3 and 4 of differentiation, respectively [27]; cardiac progenitor genes *Nkx2-5, Gata-4, Tbx5,* and *Mef2c* are expressed by day 6 [27–29]; and both alpha and beta isoforms of MyHC proteins (MyHC6/α-MyHC and MyHC7/β-MyHC, respectively) are expressed in mES cell-derived cardiomyocytes [30]. Although mES cells serve as a useful in vitro model system for studying molecular regulation of cardiomyogenesis, the roles of HH signalling during mES cardiomyogenesis have yet to be assessed.

The role of HH signalling and MEF2 factors during cardiomyogenesis in vitro has been studied in P19 embryonal carcinoma (EC) cells, a mES cell model system [31–33]. P19 cells originate from a mouse teratoma, are pluripotent, give rise to tissues in chimeric mice, and can be induced to differentiate into cardiomyocytes when treated with dimethylsulphoxide (DMSO) [34–36]. In P19 cells, overexpression of MEF2C, SHH, or GLI2 is sufficient to induce and enhance cardiomyogenesis through the upregulation of cardiac progenitor factors like *Nkx2-5* and *Gata-4* [31, 33]. In agreement, P19 cells treated with cyclopamine show delayed cardiomyogenesis [32], whereas expression of a dominant-negative GLI/EnR or *Nkx2-5*-MEF2C/EnR results in reduced cardiomyogenesis and *Nkx2*-5, *Tbx5*, *Gata-4,* and *Myhc6* expression [33]. GLI2 and MEF2C can directly bind to each other’s gene regulatory elements in P19 cells undergoing cardiomyogenesis, form a protein complex, and synergistically activate an *Nkx2-5* promoter [33]. Therefore, HH signalling and MEF2C may regulate cardiomyogenesis through a common pathway.

Chromatin remodelling factors modulate chromatin density, which affects the ability of transcription factors to regulate gene expression [37, 38]. The Brahma-associated factors (BAF) belong to the switch/sucrose non-fermentable (SWI/SNF) group of complexes and mediate nucleosome shifting on chromatin in an ATP-dependent manner [39]. When the ATPase BAF subunit, Brahma-related gene 1 (BRG1/SMARCA4) is globally knocked out, embryos do not survive past the peri-implantation stage [40]. Embryos with a conditional mutation of *Brg1* in cardiac progenitor cells, using *Nkx2-5-Cre,* have irregular ventricle morphology and die by E10.5 [41]. Therefore, BRG1 is important during heart development.

GLI3 and GLI1 proteins interact with BRG1 in the developing or postnatal brain, respectively [42]. Furthermore, BRG1 is required for both HH target gene repression and activation in mouse embryonic fibroblasts (MEFs), most probably though an interaction with GLI3R and GLI1, respectively [42], and is recruited to at least some HH target genes in a HH signalling-dependent manner [42]. Although GLI2 and BRG1 co-immunoprecipitate in MEFs, the importance of this interaction has yet to be tested [42].

Given the role of HH signalling and BAF subunits during cardiomyogenesis [18, 31–33, 41], the requirement of BRG1 for HH target gene activation, and BRG1’s ability to interact with GLI proteins [42], we hypothesized that GLI2 and BRG1 may function together to regulate cardiomyogenesis in vitro. Here we show that: 1) activation or suppression of HH signalling during mES cell cardiomyogenesis regulates cardiac progenitor transcripts *Mef2c*, *Nkx2-5*, *and Tbx5*; 2) GLI2 co-immunoprecipitates with BRG1 in differentiating mES cells; and 3) BRG1 is recruited to the GLI2 target gene, *Mef2c*, in a HH signalling-dependent manner in P19 cells undergoing cardiomyogenesis.

## Methods

### mES cell culture

D3 mES cells (ATCC, #CRL-1934) were cultured with 10 % fetal bovine serum (FBS, Wisent Inc.) and leukemia inhibitory factor (LIF, Millipore). D3 cells were stably transfected with the empty pcDNA3.1+ vector or the pcDNA3.1+ vector expressing *Flag-Gli2*^*S662A*^, a full-length complementary DNA of mouse Gli2 driven by the CMV promoter, in frame with the Flag epitope and containing the serine to alanine mutation at position 662 which prevents phosphorylation and proteasomal degradation [43]. Stable clones were termed mES[Ctrl] and mES[GLI2], respectively. Transfected colonies were selected with 0.8 mg/ml Geneticin (GIBCO) and screened for the highest FLAG-GLI2^S662A^ transcription and protein expression levels by quantitative PCR (qPCR) and western blot analysis, respectively (Additional file 1: Figure S1 and data not shown). To induce differentiation, mES cells were divided into 20 μl hanging drops at 800 cells/drop and allowed to form aggregates for two days without LIF, as described in [44]. For immunoprecipitation studies, mES cells were divided into 20 μl hanging drops at 8000 cells/drop to allow for sufficient starting material. The resulting embryoid bodies (EBs) were pooled and left in suspension for three days. Then the EBs were transferred to tissue culture-treated (TC) plates (Corning) or 0.1 % gelatin-coated (Fisher Scientific) coverslips and cultured until day 7, 10, or 15 for the analysis of cardiomyogenesis, neurogenesis, or skeletal myogenesis, respectively. Medium was changed every two days. For HH inhibition, KAAD-cyclopamine (Toronto Research Chemicals) or the methanol vehicle, were added from day 3 to day 7 at a final concentration of 3 μM where it was added every second day with medium change. KAAD-cyclopamine- and methanol-treated EBs were plated on 0.1 % gelatin-coated coverslips and were cultured until day 7.

### P19 EC cell culture

Parental P19 EC cells (ATCC, #CRL-1825) or P19 EC cells transfected with either pcDNA3-GLI2 or an empty vector control as described in [33, 45], termed P19[GLI2] and P19[Ctrl], respectively, were cultured and differentiated as per [46]. Briefly, the P19 EC cell differentiation was initiated by plating 5x10^4^ cells/ml with 1 % v/v DMSO (Sigma-Aldrich) in non-adherent dishes. After 4 days in suspension, the newly aggregated EBs were transferred to TC plates or 0.1 % gelatin-coated (Fisher Scientific) coverslips without DMSO for an additional 2 days of the 6-day protocol. Throughout the differentiation process the EBs were fed fresh medium with or without DMSO at least every two days. To inhibit HH signalling in P19 EC cells, differentiating P19 EC cultures were treated with 5 μM KAAD-cyclopamine, as previously described [32], or with methanol vehicle alone throughout the entire 6-day protocol.

### Immunoprecipitation assays

To detect FLAG-GLI2^S662A^ in the stable cell lines, total protein extracts on days 2-5 of differentiation were prepared by lysing cells in radioimmunoprecipitation (RIPA) buffer [50 mM Tris pH7.5, 150 mM NaCL, 0.2 % NP-40, 2 mM EDTA, 1X PIC (Roche), 0.5 mM PMSF (Sigma-Aldrich)]. The extracts were spun down at 17,000 x g for 30 min and the lysates were collected. 300 μg of clarified total protein lysates was submitted to immunoprecipitation with 20 μl of FLAG-beads (FLAG-IP), as per Sigma-Aldrich’s protocol. Bound proteins were eluted by boiling the beads in SDS-page sample buffer for 10 min.

For mass-spectrometric analysis, FLAG-IP was performed using 5 mg of nuclear protein extracts from day 3 differentiating mES[GLI2] and mES[Ctrl] cells as described above except that the beads with bound proteins were washed in wash buffer containing 300 mM NaCl.

For co-immunoprecipitation assay, 2 mg of total protein lysate from day 3 differentiating mES[Ctrl] and mES[Gli2] cell lines, prepared as described above, was pre-cleared for 1 h using rec-Protein-G-Sepharose beads (Invitrogen) and then were split equally and subjected to immunoprecipitation with either mouse IgG agarose beads as a negative control (Sigma-Aldrich) or FLAG beads (Sigma-Aldrich). The immunoprecipitation was performed as above except low salt wash buffer was used (100 mM NaCl, 50 mM Tris pH7.5) and the IP lasted for 2 h.

### Immunoblot analysis

The resulting IP eluates and total protein (input) samples were separated using a 4–12 % denaturing polyacrylamide gel (NuPAGE, Invitrogen) with MOPS running buffer according to the manufacturer’s protocol. The resolved proteins were transferred to a polyvinylidene fluoride (PVDF) membrane (Bio-Rad), blocked using non-fat dry milk (Carnation) reconstituted with Tris-buffered saline and Tween 20 (TBST), and incubated with GLI2- (kind gift from C.C. Hui [47]), Brg1- (Millipore), FLAG- (Sigma-Aldrich), or α-tubulin-specific (DM1A, Sigma-Aldrich) antibodies. Membranes were stripped with re-blot plus mild stripping buffer (Millipore) between each primary antibody incubation. The signal was detected using horseradish peroxidase (HRP)-conjugated secondary anti-mouse (Cell Signalling) or anti-rabbit (Santa Cruz) antibodies, followed by a chemiluminescence reaction using Pierce ECL substrate (Fisher Scientific).

Densitometry was performed on the GLI2-specific bands with the ImageJ program (National Institutes of Health, USA) [48]. The densities of the GLI2 protein in the FLAG-IP samples were normalized to the loading control, α-tubulin, and were presented as a percentage of the highest band density.

### Mass spectrometric analysis

Eluates from mES[Ctrl] and mES[Gli2] FLAG-IP, prepared as described above, were resolved on a 4–12 % denaturing polyacrylamide gel (NuPAGE, Invitrogen) and silver-stained. The staining identified a unique band profile in the 170-250 kDa range of the mES[GLI2] sample compared to the mES[Ctrl] sample (data not shown). Resolved proteins from both cell lines, within the 170-250 kDa range, were extracted from the gel using in-gel digestion, according to [49]. The extracted samples were submitted to liquid chromatography-tandem mass spectrometry (LC-MS/MS) on a Thermo LTQ Orbitrap XL hybrid mass spectrometer with a nanospray ion source. The MS/MS ion spectra were matched against the SwissProt database (version 2013_05) using the MASCOT software (Matrix Science, UK) with a peptide mass tolerance of 10 ppm and a fragment mass tolerance of 0.6 Da [50].

### Quantitative PCR (qPCR) analysis

Total RNA from mES and P19 cells was isolated using RNeasy Micro Kit (Qiagen) and E.Z.N.A. Total RNA Kit (OMEGA Bio-tek) according to manufacturers’ instructions. cDNA was generated using at least 500 ng of total RNA using the QuantiTect Reverse Transcription Kit as per manufacturer’s protocol (Qiagen). A negative control (no RT) was prepared alongside each experiment for every cell line to control against genomic DNA contamination. For each qPCR reaction, 1/40^th^ of the resultant cDNA reaction product, a final concentration of 200 nM transcript-specific primers and either a GoTaq qPCR Master Mix kit (Promega) or a KAPA SYBR® FAST qPCR kit (KAPA Biosystems) were used to detect transcripts of interest in a given sample with an Eppendorf realplex^2^ Mastercycler. The primer sequences are listed in Table 1. Threshold amplification cycles (Ct) values were determined for each sample, and normalized to the *β-actin* control using the 2^-ΔΔCt^ method [51]. The relative fold changes were presented as a percentage of the highest transcriptional expression for each respective gene (percent maximum), as described in [33, 52, 53]. All error bars represent ± standard error of the mean (SEM) from three or more independent biological replicates. All statistical analyses were done using Student’s T-tests.

**Table 1:** Sequences of primers used for real time qPCR analyses

Target	Forward Primer	Reverse Primer
*β-actin*	AAATCGTGCGTGACATCAAA	AAGGAAGGCTGGAAAAGAGC
*Ascl1*	ACTTGAACTCTATGGCGGGTT	CCAGTTGGTAAAGTCCAGCAG
*Brachyury*	CTGGACTTCGTGACGGCTG	TGACTTTGCTGAAAGACACAGG
*Brg1*	CAAAGACAAGCATATCCTAGCCA	CACGTAGTGTGTGTTAAGGACC
*Flag-Gli2* ^*S662A*^	GGACTACAAGGACGACGATGA	CAGAGGACAGGCCTTTTTCC
*Gata-4*	AAAACGGAAGCCCAAGAACCT	TGCTAGTGGCATTGCTGGAG
*Gli1*	CCAAGCCAACTTTATGTCAGGG	TCCTAAAGAAGGGCTCATGGTA
*Gli2*	CAACGCCTACTCTCCCAGAC	GAGCCTTGATGTACTGTACCAC
*Mef2c*	TCTGTCTGGCTTCAACACTG	TGGTGGTACGGTCTCTAGGA
*Mesp1*	CATCGTTCCTGTACGCAGAA	TCTAGAAGAGCCAGCATGTCG
*Myhc6*	GGGACATTGGTGCCAAGAAGA	ATTGTGGATTGGCCACAGCG
*Myhc7*	ACTGTCAACACTAAGAGGGTCA	TTGGATGATTTGATCTTCCAGGG
*Nkx2-5*	AAGCAACAGCGGTACCTGTC	GCTGTCGCTTGCACTTGTAG
*Pax3*	TTTCACCTCAGGTAATGGGACT	GAACGTCCAAGGCTTACTTTGT
*Ptch1*	AAAGAACTGCGGCAAGTTTTTG	CTTCTCCTATCTTCTGACGGGT
*Tbx5*	CTTTCGGGGCAGTGATGAC	TTGGATGAGGTGGAGAGAGC

### Immunofluorescence

Day 7 differentiated mES cells were fixed in −20 °C MeOH (Fisher), incubated 1:1 with monoclonal MF20 antibody supernatant [54] in phosphate buffered saline (PBS) and Cy3-conjugated goat anti-mouse IgG (Jackson Immuno Research) 1:100 in PBS to detect pan-MyHC expression. Coverslips were mounted in 50 parts PBS, 50 parts glycerol (Fisher), and 1 part Hoechst 33258 dye for staining nuclei. Indirect immunofluorescence of MyHC was visualized using a Leica DMI6000B inverted fluorescent microscope (Leica Microsystems GmbH) and captured with a Hamamatsu Orca AG camera (Hamamatsu Photonics). Pictures were processed with the Volocity 4.3.2 software (Perkin Elmer). Cells were counted based on the number of nuclei and identified as MyHC^+ve^ or MyHC^-ve^ using the Volocity imaging program with automated cell-identification parameters as described in ref. [44]. To measure the intensity of MHC signal, the outline of at least 10 MyHC^+ve^ individual cells in 10 random fields of view per each cell line and experiment was drawn using the software ImageJ. The intensity of the MHC staining in each outlined cell was measured, together with several background measurements. The corrected intensity values = integrated density - (cell area x mean intensity reading for the background) was calculated as in McCloy et al [55]. To assess proliferation, day 7 cells were treated with 10 μM EdU for 1 h prior to fixing with 3.7 % formaldehyde and then continuing with the EdU staining protocol (Invitrogen).

### Chromatin immunoprecipitation (ChIP) assays

ChIP assays were performed as previously described [56] using 25 μg of chromatin from day 4 differentiating P19[GLI2] or P19[Ctrl] cells and 2 μl of anti-SNF2β/BRG1 (07-478, Millipore) antibodies or Normal Rabbit Serum (NS01L, Calbiochem). Briefly, EBs from 10 plates (150 mm) were fixed with 1 % formaldehyde (Fisher Scientific) for 60 min before isolating and shearing chromatin as per [56]. The chromatin-antibody complexes were captured with BSA-blocked rec-Protein G-Sepharose 4B Conjugated beads (Invitrogen). Eluted complexes were treated with RNase A (Sigma-Aldrich) and Proteinase K (Roche), to remove contaminating RNA and proteins, respectively. The DNA was purified using a QIAquick PCR Purification Kit (Qiagen). To detect eluted DNA fragments, qPCR analyses were performed using 1/40^th^ of each eluted sample, per reaction, with sequence-specific primers listed in Table 2, as mentioned above. For every qPCR reaction, a standard curve was generated to analyze the enrichment of BRG1- or IgG-associated genomic elements from the input sample.

**Table 2:** Sequences of primers used for ChIP-qPCR analyses

Target	Location (mm10)	Forward primer	Reverse primer
*β-actin*	Chr 5:142,906,954 - 142,907,148	GATGCTGACCCTCATCCACT	ATGAAGAGTTTTGGCGATGG
Gene Desert	Chr 15: 70,644,478 - 70,644,564	TCCTCCCCATCTGTGTCATC	GGATCCATCACCATCAATAACC
*Mef2c* site A	Chr 13: 83,417,148 - 83,417,378	TGAAAAAGGAAATATCCCACTTAGA	TTGCATGGGTTCACACCTAA
*Mef2c* site B	Chr 13: 83,450,400 - 83,450,695	AGTTGCCTGAGCCTGTTTTC	TTTTTCGGCAATGATTTTCC
*Mef2c* site *C*	Chr 13: 83,517,957 - 83,518,157	CTTTCGGCTGGAGAGTCTTG	TCTCCAGTTCCTGGGAAGAA
*Mef2c* site *D*	Chr 13: 83,524,812 - 83,524,937	ACACACGCACACTTCGTCTC	GACCCACACAGAACCTTCAAA
*Mef2c* site *E*	Chr 13: 83,595,419 - 83,595,594	TTCCCATTTGGACCATTACC	ACCCACGCACTGAGACTTTC
*Mef2c* site *F*	Chr 13: 83,633,148 - 83,633,305	AACCCCAATCTTCTGCCACT	AAGCTTTCGCTAGACGTGGA
*Mef2c* site *G*	Chr 13: 83,660,831 - 83,661,075	GAGCCCCCTCTCTAATGTCC	TGTGGGCAAGTGTCTTTCTG
*Mef2c* site *H*	Chr 13: 83,664,180 - 83,664,382	AAGTGACATTTGGGGGTCCT	CGACCGACCTGCTTTACTTG
*Mef2c* site *I*	Chr 13: 83,739,543 - 83,739,715	CCTAATTATTTCAGTTTGGGATGC	CCTCCCCTCTTGTCAAAGTGT

*Chr* Chromosome

Chromatin from KAAD-cyclopamine- or MeOH-treated P19 EC cells was prepared as above with the following adapted fixing and sonication conditions. Cells from 4 plates of day 4 P19 EC EBs - treated with KAAD-cyclopamine or MeOH - were fixed with 1.5 mM Ethylene glycol bis[succinimidylsuccinate] (EGS) (Thermo Scientific) for 30 min alone, then with 1 % formaldehyde (Sigma-Aldrich) for an additional 30 min. Chromatin was sonicated in 1 mL, 12x12 AFA tubes (Covaris) for 30 min with an S220 Focused-ultrasonicator (Covaris) as per manufacturer’s recommended operating conditions for a target 200-700 bp fragment range. The IP of these samples was done using 20 μg of chromatin. The qPCR analyses were performed, as above, but with 1/50^th^ of each eluted sample, per reaction.

### Bioinformatics analysis

BRG1 genome-wide ChIP-sequencing peaks described in undifferentiated mES cells [57] were screened for overlapping conserved GLI consensus binding motifs, which were identified with the Multiple Sequence Local Alignment and Visualization tool (Mulan) as described in [58]. The nearest genes within 50 kb of these overlapping sites in the mouse genome (mm9 genome assembly) were categorized by gene ontology (GO) biological process analysis using the Genomic Regions Enrichment of Annotations Tool (GREAT) bioinformatics system as described in [59]. The entire set of BRG1 target genes in mES cells [57] was used as background for assessing the enrichment of these identified genes.

## Results

### Overexpression of FLAG-GLI2^S662A^ results in increased levels of GLI2 protein during mES cell differentiation

Since GLI2 protein is prone to degradation when phosphorylated [60], we chose to stably transfect mES cells with a *Flag-Gli2*^*S662A*^, a vector used to express a stabilized version of GLI2 [43]. qPCR showed that over the course of differentiation, transgene expression declines in all four clones analyzed (Additional file 1: Figure S1), possibly reflecting post-transcriptional down-regulation of the *Gli2* mRNA or transcript. We note that the *Gli2*^*S662A*^ mRNA that we expressed contains the entire 3’ untranslated region of the gene and as such could be subjected to microRNA-mediated downregulation [61, 62]. A similar downregulation was observed when we expressed a different transcription factor in pluripotent cells [63]. Importantly, the expression of *Gli2*^*S662A*^ mRNA persisted in all clones, albeit at lower levels throughout differentiation (Additional file 1: Figure S1). For subsequent experiments, we selected clone #3, with the highest and most stable levels of *Gli2* mRNA during differentiation (Additional file 1: Figure S1). From here on, this clone is referred to as the mES[GLI2] cell line, while control mES cells transfected with only a *Flag* peptide tag-containing vector (clone Flag#1) are referred to as the mES[Ctrl] cell line.

**Fig. 1:**
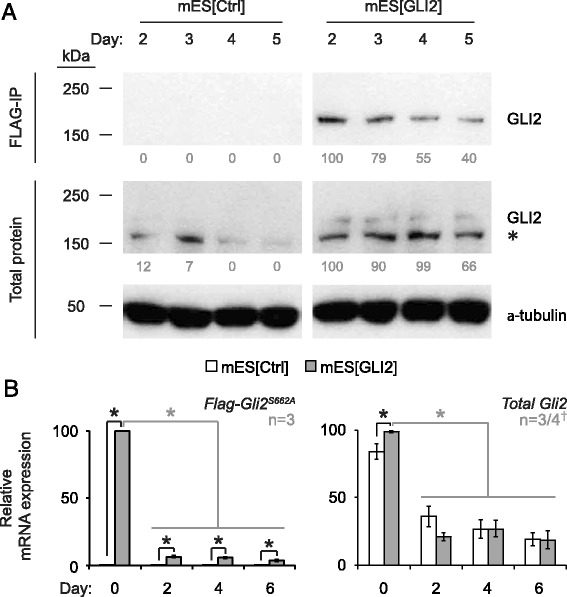
Overexpression of GLI2 protein in mES[GLI2] cultures is maintained during differentiation. **a** Total protein extracts from corresponding differentiating cells were analyzed using immunoblot with GLI2 antibodies, with or without prior FLAG-IP. α-tubulin was used as a loading control. Relative band densities are listed below blots. The size of the upper band in the total protein blots ~180 kDa, is consistent with other studies [33, 67] and equivalent to the single GLI2 band that was pulled down by FLAG-IP. *(*  *)* The non-specific binding seen below the GLI2 band is typical of this antibody [33, 47, 67]. **b** Transcription levels of the indicated genes in differentiating mES[Ctrl] (white bars) and mES[Gli2] (grey bars) cells were quantified using qPCR. Expression levels were normalized to *β-actin*, calibrated to day 0 mES[Ctrl] culture expression levels, and presented as a percentage of the highest expression level recorded, per gene. Error bars represent +/- SEM. The number of biological replicates analyzed *(*n*)* is indicated beside each graph. *(†) n* = 4 for total *Gli2* days 0 and 4. One-tailed Student’s T-tests were used for statistical analyses. Grey lines represent paired T-tests; black lines represent unpaired T-tests; *(*  *) p* < 0.05

mES[GLI2] and mES[Ctrl] cell lysates were then tested for the presence of exogenous FLAG-GLI2^S662A^ protein by immunoprecipitation with anti-FLAG beads (FLAG-IP) and sequential western blot analysis with GLI2 antibodies (Fig. 1a, first row of blots). We observed a significant enrichment of total GLI2 protein levels in mES[GLI2] cells when compared to mES[Ctrl] on days 2-5 of differentiation (Fig. 1a, second row of blots, upper band). We found ~8-fold and ~13-fold enrichment of total GLI2 protein level in mES[GLI2] cells when compared to mES[Ctrl] on days 2 and 3 of differentiation, respectively (Fig. 1a, second row of blots), and over-expression persisted on days 4 and 5, when no GLI2 protein was detectable in the mES[Ctrl] cell line at this exposure level. Overall, these results show that the GLI2 protein was overexpressed in mES[GLI2] cells during early stages of mES cell differentiation compared to the control.

Comparable results were also observed at the transcriptional level. *Flag-Gli2*^*S662A*^ transcripts were only expressed in the mES[GLI2] cells and were at the highest level in the undifferentiated (day 0) mES[GLI2] cells (Fig. 1b, panel *Flag-Gli2*^*S662A*^), and this was mirrored by total *Gli2* transcript levels (Fig. 1b and Additional file 1: Figure S1). Such a trend has been noted in other published work on differentiating mES cells [33]. Thus, the stability of the exogenous GLI2^S662A^ protein, which remains higher during the differentiation of mES[GLI2] cells compared to controls, is a key element in our model system.

### Overexpression of GLI2 results in enhanced cardiac progenitor gene expression

**Fig. 2:**
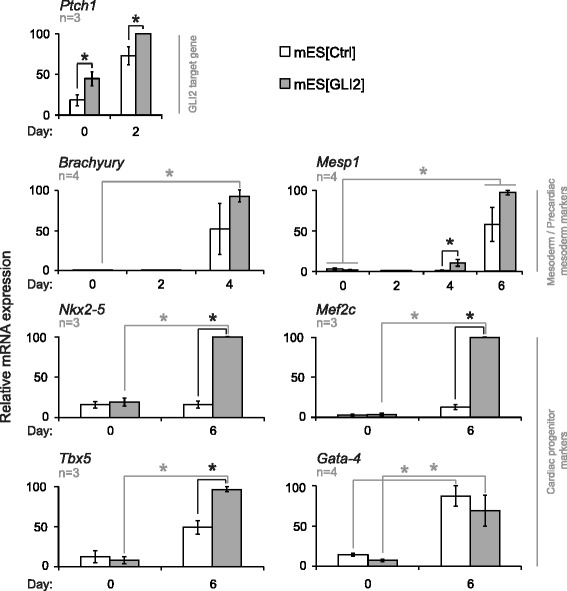
Overexpression of GLI2 in mES cells enhances transcript expression of a GLI2 target and of cardiac progenitor-enriched genes. qPCR analysis of mRNA expression of indicated genes known to be active in HH-responsive cells, mesoderm/precardiac mesoderm cells, and cardiac progenitor cells, in mES[Ctrl] (white bars) and mES[Gli2] (grey bars) cultures on the days noted in the figure. Expression levels were normalized to *β-actin*, calibrated to day 0 mES[Ctrl] culture expression levels, and presented as a percentage of the highest expression level recorded, per gene. Error bars represent +/- SEM. The number of biological replicates analyzed *(*n*)* is indicated on each graph. One-tailed Student’s T-tests were used for statistical analyses. Grey lines represent paired T-tests; black lines represent unpaired T-tests; *(*  *) p* < 0.05

To assess the efficacy of the GLI2 overexpression, we monitored the expression of the GLI2 target gene, *Ptch1* [64]. Overexpression of GLI2 resulted in a significant 2.9 ± 0.7-fold increase of *Ptch1* transcripts in undifferentiated mES[GLI2] cells and a significant 1.4 ± 0.2-fold increase in day 2 differentiating mES[GLI2] cells compared to control cells (Fig. 2, panel *Ptch1*). Therefore, the overexpression of GLI2 in mES cells increased GLI2 target gene expression.

During differentiation, both the mES[Ctrl] and mES[GLI2] cell lines showed a similar transition through the mesoderm stage of differentiation, as no significant difference was detected for the levels of *Brachyury* transcripts by day 4 between these two cell lines (Fig. 2, panel *Brachyury*). *Mesp1* levels were significantly upregulated in the mES[GLI2] cultures as compared to control cultures on day 4 of differentiation, however, its level of expression was minor as compared to day 6 values and was not significantly higher than day 0 basal levels (Fig. 2, panel *Mesp1*). On day 6, no significant difference between mES[Ctrl] and mES[GLI2] cell lines was detected for the *Mesp1* expression. Based on this analysis, the mesoderm and precardiac mesoderm stages of cardiomyogenesis during mES cell differentiation did not appear to be significantly regulated by GLI2 overexpression. These results are supported by previous reports, which showed that modulation of HH signalling in mouse P19 EC cells did not significantly regulate mesoderm induction [31–33].

The cardiac progenitor transcripts *Nkx2-5*, *Mef2c*, and *Tbx5* were significantly upregulated in the mES[GLI2] cultures on day 6 of differentiation as compared to mES[Ctrl] cells (Fig. 2; panels *Nkx2-5*, *Mef2c*, and *Tbx5*). Interestingly, overexpression of GLI2 had no effect on day 6 *Gata-4* transcript levels (Fig. 2, panel *Gata-4*). Therefore, overexpression of GLI2 during mES cell differentiation resulted in enhanced transcription levels of select cardiac progenitor genes. Our data supports and extends previous publications, where overexpression of GLI2 was shown to induce and enhance cardiac progenitor gene expression in P19 EC cells [31, 33].

We tested whether cells of the skeletal muscle and neuronal lineages were affected, since GLI2 and HH have been shown to regulate these processes [14, 65, 66]. However, we did not detect any significant increase in the mRNA levels of Pax3 (Additional file 2: Figures S2A), a gene involved in skeletal myogenesis and downstream of GLI2 [45, 53, 67, 68], or in MyHC-positive skeletal myocytes (data not shown as none of the cultures contained any skeletal myocytes after 10 days of differentiation). A significant increase in Ascl1, a gene involved in neural progenitor differentiation that is downstream of GLI2 and HH signalling [67, 69–71], was detected (Additional file 2: Figure S2B). However, none of the cultures contained NF68-positive neurons, even after 10 days of differentiation (negative data not shown).

### Overexpression of GLI2 does not result in an increased number of cardiomyocytes

**Fig. 3:**
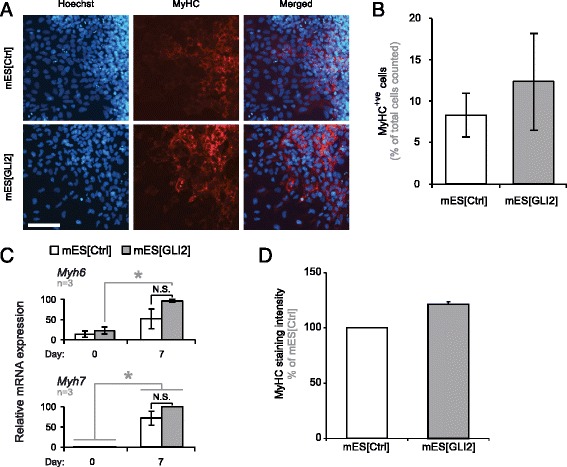
Overexpression of GLI2 does not significantly upregulate the number of MyHC^+ve^ cells. **a** MyHC^+ve^ cells *(red)* were visualized and (**b**) counted in corresponding day 7 differentiating cells by indirect immunofluorescence. Hoechst *(blue)* was used to visualize nuclei. Representative images of the cardiomyocyte-enriched areas on the periphery of an EB are shown. Scale bar represents 100 μm. At least 2500 nuclei were counted across 20 random fields of view, per biological replicate; *n* = 3. **c** *Myhc6/7* expression levels on the days indicated in mES[Ctrl] (white bars) and mES[Gli2] (grey bars) cells were normalized to *β-actin*, calibrated to day 0 mES[Ctrl] culture expression levels, and presented as a percentage of the highest expression level recorded, per gene. Error bars represent +/- SEM; *n* = 3. One-tailed Student’s T-tests were used for statistical analyses. Grey lines represent paired T-tests; black lines represent unpaired T-tests; *(*  *) p* < 0.05. **d** MyHC^+ve^ cells from (**a**) were analyzed for MyHC signal intensity using the ImageJ program. At least 100 cells were analyzed across 10 random fields of view per biological replicate; *n* = 2

To determine if the enhancement of select cardiac progenitor transcripts led to an overall enhancement of cardiomyocytes in mES[GLI2] cultures, we counted MyHC^+ve^ cells in day 7 mES[Ctrl] and mES[GLI2] cultures (Fig. 3a). On average, 8.3 ± 2.6 % and 12.4 ± 5.9 % of the total cells counted were MyHC^+ve^ in the mES[Ctrl] and mES[GLI2] cultures, respectively (Fig. 3b). Similar percentages have been shown before by analysis of MyHC, MLC2v and tropomyosin in day 7 or 8 differentiating mES cells, differentiated using the hanging drop method [44, 72]. Although there was a trend towards an increase in cardiomyocytes in the mES[GLI2] cultures compared to the control, the variability between the samples across three biological replicates (Fig. 3b) and another four additional replicates (data not shown) resulted in no significant difference in the percentage of MyHC^+ve^ cells on day 7.

Although overexpression of GLI2 during mES cell differentiation also did not significantly affect the level of *Myh6* or *Myh7* transcripts on day 7 (Fig. 3c), we observed a notable increase in MyHC signal intensity in cardiac myocytes in mES[GLI2] cultures (Fig. 3d). This suggests that although the number of cardiomyocytes formed is unchanged, the presence of exogenous GLI2 is associated with a trend towards higher MyHC protein expression.

To test if overexpression of GLI2 had any effect on cell proliferation or apoptosis, we first analyzed EdU-positive cells. When cultures were analyzed for the presence of EdU-positive cells on day 7, there was no difference between mES[Ctrl] and mES[Gli2] cell lines (Additional file 3: Figure S3A). Of note, we also did not observe any differences in RNA yields between mES[Ctrl] and mES[Gli2] cultures throughout the 7-day differentiation protocol (Additional file 3: Figure S3B). This supports that overexpression of GLI2 did not have any overt effects on cell proliferation. Lastly, we analyzed cells with fragmented nuclei as a classical hallmark of apoptosis. Notably, there was no difference in the proportion of apoptotic cells in mES[Gli2] cultures when compared to mES[Ctrl] (Additional file 3: Figure S3C). Thus, overexpression of GLI2 did not have any effect on cell proliferation or survival in differentiating mES cells.

### Antagonizing the HH pathway leads to lower cardiac progenitor gene expression

**Fig. 4:**
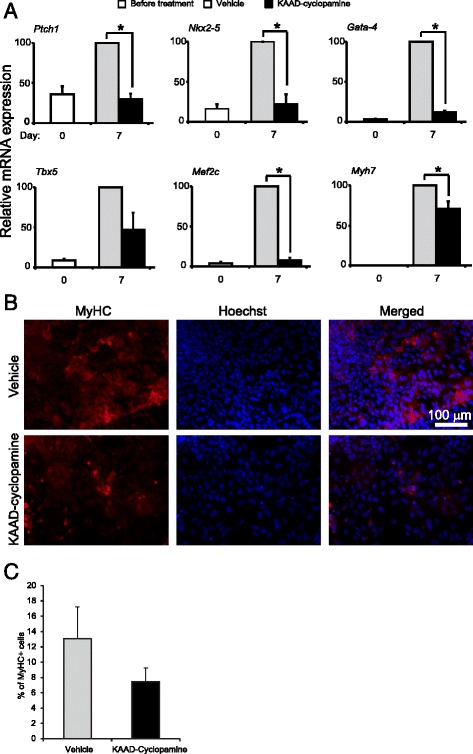
HH signalling blockade prevents the induction of GLI2 target and of cardiac progenitor-enriched genes. **a** qPCR analysis of mRNA expression of Ptch1 and indicated cardiac progenitor-enriched genes in day 0 mES cells (white bars) or in cultures kept in differentiation condition for 7 days, in the presence of KAAD-cyclopamine (black bars) or vehicle (methanol, grey bars). Error bars represent +/- SEM; *n* = 3 *(*  *) p* < 0.05 by one-tailed paired *T*-test. **b** Immunofluorescence detection of myosin heavy chain (red channel) in similar day 7 cultures treated with vehicle or KAAD-cyclopamine. DNA was counterstained with Hoechst (blue channel). **c** MyHC^+ve^ cells from (**b**) were counted. Error bars represent +/- SEM; *n* = 3 biological replicates

A previous report demonstrated active HH signalling during mES differentiation [33]. Our results showing induced levels of Ptch1 expression, a readout of active HH signalling, during mES(Ctrl) differentiation (Fig. 2, panel Ptch1), are in line with this observation. Thus, following the results from gain-of-function experiments, we proceeded to investigate the involvement of GLI2 through loss-of-function experiments. For this, we utilized a synthetic derivative of cyclopamine, KAAD-cyclopamine, which is approximately 10-20 times more potent than cyclopamine without being more toxic [73] and has previously been shown to successfully inhibit HH signalling in a variety of cells, such as P19 EC cells, C3H10T1/2 fibroblasts, and adult skeletal muscle satellite cells [32, 53]. HH inhibition was monitored by the downregulation of *Ptch1* mRNA, a marker of active HH signalling [74]. KAAD-cyclopamine treatment resulted in a 69 ± 6 % decrease in *Ptch1* expression on day 7 of mES cell differentiation, as compared to vehicle-treated cells (Fig. 4a). HH inhibition also resulted in comparable decreases in expression of *Nkx2-5*, *Gata-4*, *Tbx5* and *Mef2c* (Fig. 4a). We also noted a significant, albeit more modest, decrease in *Myh7* expression (Fig. 4a). By immunofluorescence, we noted a slight, not statistically significant decrease in the number of myosin heavy chain-positive cells (Fig. 4b and c). To rule out that the observed effect on MyHC cardiac myocytes was not due to aberrant cell proliferation or apoptosis, we first tested the RNA yields from identical plates of vehicle and cyclopamine treated cultures. Throughout 7-day differentiation protocol, there was no difference in the amount of RNA harvested from vehicle-control and cyclopamine treated cultures (Additional file 3: Figure S3D), suggesting that there was no overt effect on cell proliferation in the presence of cyclopamine. To test for aberrant apoptosis, cells with fragmented nuclei were analyzed (Additional file 3: Figure S3E). These controls rule out the possibility that cell density or survival differences elicited by cyclopamine treatment would have caused the phenotype we observed. Together, our results on HH activation (GLI2 overexpression, Fig. 2) and inhibition (KAAD-cyclopamine treatment, Fig. 4) support the notion that HH signaling is important for cardiac progenitor gene expression, similarly to previous reports in P19 cells [31, 32].

### GLI2 interacts with BRG1 during mES cell differentiation

**Fig. 5:**
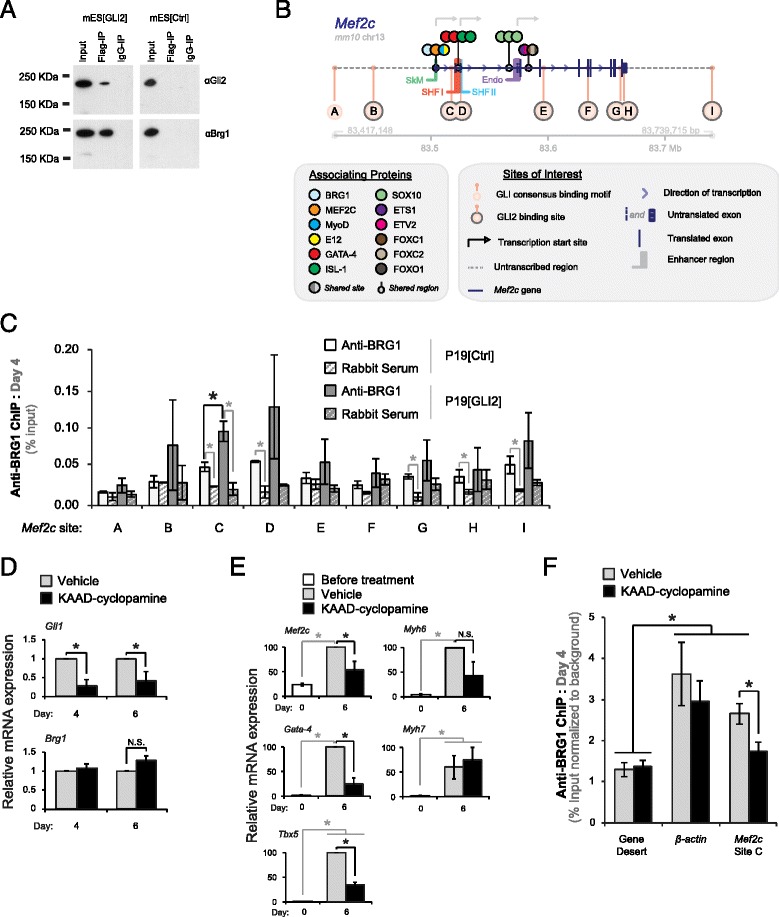
Overexpression of GLI2 recruits BRG1 to a GLI2-specific *Mef2c* gene element. **a** Anti-Flag immunoprecipitation in nuclear extracts from day 3 mES[GLI2] or mES[Ctrl] cells. Immunoprecipitated proteins were probed by western blot using anti-BRG1 or anti-GLI2 antibodies. **b** A schematic representation of the *Mef2c* gene and its major sites of interest. The scale bar represents coordinates of the *Mef2c* gene in the mouse genome (mm10 genome assembly). This schematic was constructed using the UCSC Genome Browser (http://genome.ucsc.edu), TRANSFAC, and data from previous publications [33, 82–84, 94, 96–101]. GLI-specific *Mef2c* sites *A-I* are marked with beige circles. GLI2 has been shown to bind only sites *B-I (circles outlined in grey)*. Other associating proteins, including BRG1 and MyoD, are depicted with coloured circles as outlined in the legend. A detailed description of the associating protein sites can be found in Table 3. *Chr:* Chromosome. *SHF I*: ISL-1-dependent SHF enhancer. *SHF II*: NKX2-5/FOXH1-dependent SHF enhancer. **c** Anti-BRG1 ChIP was performed on day 4 differentiating P19[GLI2] and P19[Ctrl] cultures with sequential qPCR analyses of GLI-specific *Mef2c* sites *A-I,* depicted in *(B)*. One-tailed Student’s T-tests were used for the ChIP statistical analyses; *n* = 3. All error bars represent +/- SEM. All grey lines represent paired T-tests; all black lines represent unpaired T-tests; *(*  *) p* < 0.05. **d** *Gli1* and *Brg1* mRNA expression levels were assessed by qPCR in differentiating P19 EC cultures, treated with MeOH vehicle or KAAD-cyclopamine. These levels were normalized to *β-actin* and presented as a fold-change over MeOH-treated culture expression levels from the same day. **e** The effect of KAAD-cyclopamine (black bars) or vehicle (grey bars) treatment on the expression level of Gli1 and indicated cardiomyogenesis-specific genes was assessed using qPCR analysis. Expression levels were normalized to *β-actin*, calibrated to day 0 untreated culture expression levels, and presented as a percentage of the highest expression level recorded, per gene. **f** Anti-BRG1 ChIP was performed on day 4 differentiating P19 EC cultures that were treated with either MeOH vehicle (grey bars) or KAAD-cyclopamine (black bars). Each ChIP was followed by qPCR analyses on a gene desert region *(negative control)*, *β-actin (positive control)*, and *Mef2C* site *C* from panel **b**. All error bars represent +/- SEM; *n* = 3. Two-tailed Student’s T-tests were used for statistical analyses. All grey lines represent paired T-tests; all black lines represent unpaired T-tests; *(*  *) p* < 0.05

We further sought to elucidate the molecular mechanisms behind the enhanced *Mef2c, Nkx2-5*, and *Tbx5* transcription levels on day 6 of mES cell differentiation by GLI2 overexpression. To identify potential GLI2-interacting proteins, we performed a mass spectrometric and western blot analysis of the FLAG-IP on mES[GLI2] and mES[Ctrl] cell nuclear extracts from day 3 differentiating cells. BRG1, an active member of the BAF chromatin-remodelling complex was identified and had a higher Mascot score in the mES[GLI2] sample than the mES[Ctrl] sample, in the mass spectrometry assay (data not shown). A sequential western blot assay with anti-GLI2 antibodies confirmed immunoprecipitation of GLI2 protein in mES[GLI2], but not in mES[Ctrl] cells (Fig. 5a). When the same blot was re-probed with anti-BRG1 antibodies, co-immunoprecipitation of BRG1 with FLAG-GLI2^S662A^ was observed (Fig. 5a). The co-IP BRG1 signal in the mES[GLI2] cells was not merely due to modulated levels of *Brg1* expression, as both mES[Ctrl] and mES[GLI2] samples showed no significant difference in *Brg1* mRNA transcript levels during the first four days of differentiation (Additional file 3: Figure S3A). Our results showing co-precipitation of BRG1 with GLI2 are similar to those reported by others using NIH 3 T3 cells, where BRG1 was found to interact with HA-tagged GLI proteins, including GLI2 [42].

The observed co-immunoprecipitation of GLI2 and BRG1 during mES cell differentiation led to our hypothesis that GLI2 could recruit BRG1 to regulatory regions of GLI2 target genes to modulate their expression. Given the results and the known roles of BRG1 and GLI2 in heart development [19, 20, 41], we were interested in knowing if GLI2 and BRG1 could co-regulate key cardiac progenitor genes implicated in cardiomyogenesis. *Mef2c*, which is important for proper mammalian heart development [8, 75], was enhanced in day 6 differentiating mES[GLI2] cells compared to mES[Ctrl] cells (Fig. 2) and was recently identified as a direct target of GLI2 during P19 EC cell cardiomyogenesis [33]. Thus, we set out to determine if BRG1 associates with GLI2-bound *Mef2c* regulatory elements in a HH-dependent manner. Since mES cells spontaneously differentiate into lineages of all three germ layers [76] and BRG1 is expressed in many mammalian cells and tissues [39], it is difficult to answer this question in the context of endogenous mES cell cardiomyogenesis. In contrast, P19 EC cells predominantly differentiate into cardiomyocytes by day 6 of differentiation, along with skeletal myogenic progenitors and fibroblast-like cells when treated with DMSO [35, 77, 78]. Moreover, P19 EC differentiation is very similar to mES myogenic differentiation [33, 44, 79, 80]. For these reasons, we used P19 cells to determine the role of BRG1-GLI2 protein complex during myogenic differentiation.

### BRG1 associates with *Mef2c* regulatory elements in HH signalling dependent manner

In line with what has been shown previously [81], we determined that *Brg1* is expressed throughout the differentiation of mES and P19 cells, and that GLI2 over-expression does not drastically affect its expression in either (Additional file 4: Figure S4). Our lab has previously shown that GLI2 associates with eight of the nine conserved GLI consensus binding motifs found in the *Mef2c* gene on day 4 of P19[GLI2] cell differentiation (*Mef2c* sites *B-I*) (Fig. 5b and Table 3) [33]. Given that BRG1 immunoprecipitates with GLI2, we performed an anti-BRG1 ChIP on chromatin from day 4 differentiating P19[Ctrl] and P19[GLI2] cells, to determine if BRG1 associates with GLI2-specific *Mef2c* genomic elements during differentiation, and also if GLI2 overexpression can modulate this potential association. Notably, P19[GLI2] cells exhibited enhanced cardiomyogenesis as measured by an increased percentage of cardiomyocytes and higher expression levels of cardiac progenitor genes on day 6 of differentiation, in agreement with previous reports (references [31, 33] and data not shown).

**Table 3:** A selection of proteins that associate with the *Mef2c* gene

Protein	*Mef2c* domain	Target Sequence^a^	Location (mm10: Chr 13)	Source of Protein	References^b^
BRG1	-	-	83.504,012 - 83,504,138	Mouse	[84]
MEF2C	MADS-box	acctttacagCTAAATTTACtccagagtg	83,504,087 - 83,504,115	Mouse	[94]
MyoD	E-box	gagtgacatgaaCAGGTGcaccctggcct	83,504,111 - 83,504,139	Mouse	[94]
E12	E-box	gagtgacatgaaCAGGTGcaccctggcct	83,504,111 - 83,504,139	Mouse	[94]
GATA-4	GATA-d	taagagttcTTATCAgtgtc	83,523,287 - 83,523,306	Rat	[82]
	GATA-p	gtcacccgctatCTATCGgtcagg	83,523,351 - 83,523,374		
ISL-1	ISL-d	gtcaggggagcCTAATGcatttgggaa	83,523,369 - 83,523,395	Hamster	[82]
	ISL-p	ggtttacttgCTAATGacctggataa	83,523,405 - 83,523,430		
SOX10	SOX	gaatgcactgacTACAAAGtgcatcctgaag	83,565,883 - 83,565,913	Mouse	[96]
	Binding	ggccatttagctCACAATGaaggtctgtgtt	83,565,913 - 83,565,943		
	Site	aaatagctctatAACAAAGtaactacagagt	83,565,947 - 83,565,977		
ETS1	ETS-A	agttactcTCTTCCTGttatgaca	83,582,754 - 83,582,777	Mammalian	[97, 98]
ETV2	ETS-A	agttactcTCTTCCTGttatgaca	83,582,754 - 83,582,777	Mammalian	[97]
FOXC1	FOX-NC	ggaagttactctcttccTGTTATGacaggaaagcgtagaca	83,582,752 - 83,582,791	Mouse	[97]
FOXC2	FOX-NC	ggaagttactctcttccTGTTATGacaggaaagcgtagaca	83,582,752 - 83,582,791	Mouse	[97]
FOXO1	FOX-NC	ggaagttactctcttccTGTTATGacaggaaagcgtagaca	83,582,752 - 83,582,791	Mouse	[97]

*Chr* Chromosome

^a^Uppercase nucleotides in the target sequence represent nonconsensus binding domains

^b^All binding site information for proteins, except BRG1 and SOX10, were compiled with information gathered through TRANSFAC

Results from the anti-BRG1 ChIP showed an association of BRG1 with *Mef2c* sites *C, D,* and *G-I* in the P19[Ctrl] cells as compared to ChIP with a non-specific rabbit serum control (Fig. 5c). Notably, although there was a similar trend in the P19[GLI2] cells, we did not observe statistical significance for BRG1 association on sites *D* and *G-I* when compared to non-specific rabbit serum (Fig. 5c). *Mef2c* site *A*, which lacked GLI2 association under the same experimental conditions in a previous study [33], showed no significant association with BRG1 in either cell line (Fig. 5c). However, the association of BRG1 with the *Mef2c* site *C* was significantly (*p* < 0.05) higher in P19[GLI2] cells as compared to P19[Ctrl] cells (Fig. 5c). This effect was not due to altered BRG1 expression in P19[GLI2] cells as there was no significant change in *Brg1* expression in P19[GLI2] cells, as compared to P19[Ctrl] cells, on day 4 of differentiation, when the ChIP assay was performed (Additional file 4: Figure S4B).

*Mef2c* site *C* is of significant interest as it is located proximally to the ISL-1-dependent SHF enhancer region [82] (Fig. 5b, SHF1), it is upstream of the start site of a transcript expressed in the developing heart [83], and it is also the closest GLI2-binding site to a region previously shown to associate with BRG1 [33, 84] (Fig. 5b, light blue circle). Thus, as overexpression of GLI2 resulted in increased association of BRG1 with *Mef2c* site *C*, we chose this site to further investigate the dependence of BRG1 association on endogenous HH signalling during P19 EC cell cardiomyogenesis. KAAD-cyclopamine treatment of P19 EC cells resulted in a 71.7 ± 17.0 % and 60.5 ± 25.0 % decrease in *Gli1* expression on days 4 and 6 of P19 EC cell differentiation, respectively, without significantly affecting the day 4 or 6 *Brg1* transcript levels, compared to treatment with a vehicle control (Fig. 5d). Similar cyclopamine-mediated reductions of *Gli1* expression have been seen before in P19, P19CL6, and mES cells [32, 85, 86]. By day 6, differentiating P19 EC cells treated with KAAD-cyclopamine expressed significantly lower *Mef2c* (45.9 ± 16.7 %), *Gata-4* (74.8 ± 12.2 %), and *Tbx5* (65.4 ± 5.9 %) transcript levels, compared to differentiating cells treated with the vehicle control (Fig. 5e; panels *Mef2c*, *Gata-4*, and *Tbx5*). These lower levels of cardiac progenitor-enriched transcripts were expected as cyclopamine-treated P19 EC cells have repressed *Gata-4* expression [32] and cyclopamine-treated P19CL6 EC cells show lower levels of *Gata-4* and *Nkx2-5* transcripts [85] and we showed that a similar treatment in mES cells has a comparable effect (Fig. 4). Neither the level of contractile protein transcripts in the day 6 differentiated cells (Fig. 5e, panels *Myhc6* and *Myhc7*) nor the number of MyHC^+ve^ cardiomyocytes (data not shown) significantly differed between KAAD-cyclopamine and vehicle-treated P19 EC cells. This is supported by previous reports, where differentiating P19 EC cells treated with cyclopamine did not show any difference in the number of MyHC^+ve^ cardiomyocytes [32]*.* Thus, inhibition of HH signalling downregulated, but did not abolish, normal expression of cardiac progenitor specific transcripts and, overall, did not repress the formation of cardiomyocytes, in agreement with [32].

To test if inhibition of HH signalling attenuated the ability of BRG1 to associate with *Mef2c* site *C*, we performed an anti-BRG1 ChIP in day 4 differentiating P19 EC cells treated with vehicle or KAAD-cyclopamine (Fig. 5f). The association of BRG1 with *Mef2c* site *C* in P19 EC cells was significantly reduced upon HH inhibition (Fig. 5f). Importantly, KAAD-cyclopamine treatment had no significant effect on BRG1’s association with *β-actin* a known target of BRG1 in mES cells [57]. A gene desert locus was used as a negative control and showed no significant association with BRG1. Therefore, HH signalling is required, at least to some extent, for the efficient association of BRG1 to the GLI2-specific *Mef2c* gene element *C*.

## Discussion

**Fig. 6:**
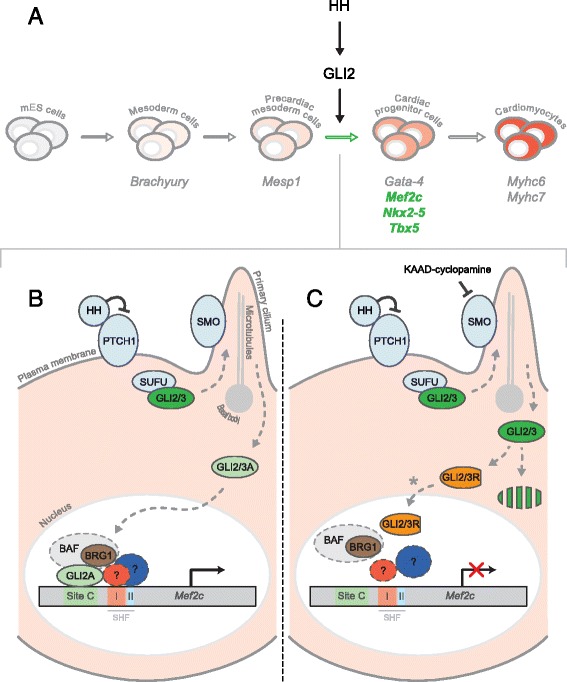
A model summarizing the role of the HH signalling pathway and its primary transducer, GLI2, during mES cell cardiomyogenesis. **a** In this study, GLI2 expression was observed to positively regulate cardiac progenitor-enriched genes in mES cells *(highlighted in green)*. The solid black arrows indicate HH/GLI2’s proposed direct regulation. The hollow green arrow marks the transition that is enhanced by GLI2. **b** GLI2-regulated enrichment of cardiac progenitor transcripts may be explained in part by the ability of the transcriptional activator form of GLI2 (GLI2A) *(light green ellipses, B)* - mediated via a functional HH signalling pathway *(light blue ellipses)* - to enrich BRG1 association at GLI2-specific *Mef2c* site *C*. This enrichment site is proximal to known SHF enhancer regions *(I,II)*. Other cofactors *(dash-outlined ellipses)*, including the remaining BAF complex members and other unidentified SHF-related transcription factors *(?)* may support this GLI2-mediated association. **c** When KAAD-cyclopamine inhibits HH signalling, GLI2 is likely degraded, which leads to a reduction of BRG1 association on *Mef2c* site *C*. Although we have not observed the formation of GLI2R in our system (data not shown), it is possible that GLI2 may be processed into GLI2R *(orange truncated ellipses, C)* when HH signalling is blocked [13]*.* Also, GLI3R *(included in the orange truncated ellipses, C)* may contribute to the repression of *Mef2c* expression. The downstream binding of either GLI2R or GLI3R to *Mef2c* has yet to be assessed *(*  *)*

In this study we have shown that 1) GLI2 enhances the expression of cardiac progenitor-enriched genes while blocking HH signalling through cyclopamine decreases the expression of cardiac progenitor-enriched genes; 2) GLI2 immunoprecipitates with BRG1 during mES cell cardiomyogenesis; and 3) BRG1 is recruited to a GLI2-specific *Mef2c* gene element in a HH-dependent manner. Thus, we propose a model, where HH/GLI2 regulates the expression of *Mef2c* via the recruitment of BRG1 to the *Mef2c* gene element upstream of the SHF elements to ultimately regulate cardiomyogenesis in stem cells (Fig. 6).

### HH signalling is important for cardiac progenitor gene expression during mES cell differentiation

Using a stabilized GLI2 mutant, we have shown that GLI2 enhances early expression of cardiac progenitor transcripts such as *Mef2c*, *Nkx2-5*, and *Tbx5* during mES cell cardiomyogenesis (Fig. 2). In agreement, when HH signalling in mES cells is blocked with cyclopamine, this leads to a great reduction in the expression of these genes (Fig. 4). This supports and expands previous reports in P19 EC cells [32, 33].

The effect of cardiac progenitor gene upregulation by exogenous Gli2 is probably not due to an increase in mesodermal transcript expression as GLI2 overexpression does not have any significant effect on the levels of *Brachyury* or *Mesp1* on days 4 and 6, respectively (Fig. 2). Notably, the small upregulation of *Mesp1* transcript levels on day 4 in mES[GLI2] cells is minor in comparison with day 6 values. This is in agreement with previous studies, which demonstrate that there is no significant HH-dependent effect on mesoderm transcripts in P19 EC cells [31–33].

The enhanced expression of *Nkx2-5* in mES[GLI2] cells correlates with the HH-mediated expression of *Nkx2-5* in vivo [4, 18] and in P19 EC cells [31–33]. The upregulation of *Tbx5* transcript levels in differentiating mES[GLI2] cells (Fig. 2) follows the same pattern of *Tbx5* upregulation seen in differentiating P19[GLI2] cells [33]. When HH signalling is inhibited in P19 EC or mES cells by KAAD-cyclopamine, *Tbx5* expression is reduced (Figs. 4 and 5e), similarly to P19 EC cells overexpressing dominant-negative GLI/EnR [33]. Interestingly, Tbx5 is expressed in SHF cells that receive HH signals in vivo*,* and a TBX5-HH network is required for atrial septation [87]. Although the regulation of *Tbx5* expression by the HH pathway is yet to be determined in vivo, based on results from previous reports and our observations, *Tbx5* expression is regulated by HH signalling in vitro*,* during P19 EC and mES cell differentiation.

In contrast to *Mef2c, Nkx2-5*, and *Tbx5* upregulation in mES[GLI2] cells, *Gata-4* expression is not affected when GLI2 is overexpressed (Fig. 2). Importantly, *Gata-4* transcript levels were downregulated in mES cells treated with cyclopamine (Fig. 4), similarly to P19 EC cells (Fig. 5e and ref. [32]). This suggests that while exogenous Gli2 cannot enhance *Gata-4* expression in differentiating mES cells, repression of endogenous HH signalling is sufficient to reduce its expression. It is possible that *Gata-4* induction is more efficient with other Gli family members, that *Gata-4* responds to Hh signaling with a different kinetic, or that it responds at a different threshold of Hh-Gli activity, compared to the other cardiac transcription factor genes. Previous studies have shown that *Gata-4* expression might be independently regulated from *Mef2c*, *Nkx2-5*, and/or *Tbx5* expression [84, 88, 89]. Removal of the adjacent endoderm from the side of a developing avian heart, or a conditional knockout of *Baf250a* in the SHF of the developing murine heart results in decreased *Nkx2-5* and *Mef2c* expression while *Gata-4* levels remain unaffected [84, 88]. In mES cells, overexpression of the cardiac progenitor gene regulator, Yin Yang 1 (YY1) enhances *Nkx2-5* and *Tbx5* expression with no apparent effect on *Gata-4* expression [89]. Therefore, our observation of *Mef2c*, *Nkx2-5*, and *Tbx5* upregulation in mES[GLI2] cells with no observable effect on day 6 *Gata-4* expression is in line with previous studies and could provide insight into a Gli2-dependent and independent regulation of mES cell cardiomyogenesis.

In summary, the activation or suppression of the HH signaling in mES cells yields a concerted up- or down-regulation of multiple cardiac progenitor transcription factor genes. Since our experiments were carried out using total cell populations, we cannot rule out the possibility that this occurred in a disorganized manner, with individual cells upregulating only one of the multiple cardiac progenitor specific genes we saw induced at the population level. Previous work by our and other labs, as well as this work, demonstrate that the induction patterns of Nkx2-5, Tbx5, MEF2C and Gata-4 in differentiating mES and P19 cells mimic each other, ultimately culminating in maximal transcription on the same days of differentiation, just before or on the onset of the expression of structural cardiac muscle genes (Fig. 2, and [27–29, 32, 33, 45]). Moreover, simultaneous expression of Gata-4 and Tbx5 is detected in hES-derived cardiovascular progenitors and cardiomyocytes, but not in smooth muscle or endothelial cells [90]. These reports suggest that the expression of multiple signature cardiac muscle progenitor genes represents an induction of cardiac muscle progenitors and differentiated cardiomyocytes. Therefore, we deem unlikely the possibility of illegitimate expression of the overall signature cardiac progenitor specific genes in other cell types. Nevertheless, a transcriptome analysis at a single-cell level or after enrichment using cell surface markers specific for cardiac progenitors would help to directly address this question by providing an estimate of cell heterogeneity in the cultures.

### Modulation of HH signalling does not alter the number of mES cardiomyocytes

Although modulation of HH signalling through Gli2 overexpression or application of cyclopamine regulates *Nkx2-5*, *Mef2c* and *Tbx5* cardiac progenitor gene expression in mES cells (Figs. 2 and 4), this does not alter the percentage of cardiomyocytes (Figs. 3 and 4). One explanation might lie in *Gata-4* expression. Namely, *Gata-4* appears to be a limiting factor, in a transcription factor cocktail, to induce MyHC expression in mES cells [91]. Therefore, the lack of *Gata-4* enhancement by exogenous GLI2 may offer an explanation for the lack of cardiomyocyte number increase in mES[GLI2] cultures. HH signalling gain-of-function experiments in P19 cells showed both an induction and enhancement of cardiomyogenesis [31, 33]. P19 cells may be more susceptible to differentiate into cardiomyocytes as they are more prone to differentiate into cells of the mesodermal lineage [35]. In contrast, mES cells differentiating through EBs in the absence of any other external stimuli give rise to cell types from all three germ layers [76, 92, 93] and thus may not have as many mesoderm cells that are susceptible to HH-mediated regulation. It is possible that GLI2 overexpression affects the differentiation of other cell types as HH signalling regulates many developmental programs in vivo [14, 65, 66], resulting in a lower percentage of cardiomyocytes in mES[GLI2] cultures. Another possibility is that there may be other additional factors, mechanisms and signalling pathways in mES cells that regulate the transition of pre-cardiac mesoderm to cardiomyocyte progenitor cell when compared to P19 EC cells. Lastly, HH signalling may enhance mechanisms in the neighbouring lineages that in turn negatively regulate cardiac differentiation in mES cells.

### GLI2 immunoprecipitates with BRG1 during mES cardiomyogenesis and active HH signalling regulates BRG1 association with *Mef2c* gene

To determine the mechanism of Gli2 during in vitro cardiomyogenesis, we used both mES(Gli2) and P19(Gli2) differentiating cells. The co-immunoprecipitation of GLI2 and BRG1 in differentiating mES cells (Fig. 5a) is in agreement with previous observations of HA-tagged GLI2 immunoprecipitation with BRG1 in NIH 3 T3 cells [42]. To investigate the significance of this interaction, we tested the hypothesis that GLI2 and BRG1 co-regulate cardiomyogenesis in vitro by modulating the expression of GLI2 target genes. In this light, we focused on *Mef2c* gene, a direct target of GLI2 during P19 EC cell cardiomyogenesis [33]. Increased *Mef2c* transcript levels in mES[GLI2] (Fig. 2) support and extend these results. Moreover, BRG1 associates with the first exon in the *Mef2c* gene [84], where other proteins, including MyoD, are known to bind [94], but which lacks GLI consensus binding site (Fig. 5b).

The anti-BRG1 ChIP analysis from day 4 differentiating P19 EC control cells demonstrates that BRG1 associates with *Mef2c* sites *C*, *D*, and *G-I* (Fig. 5c), which GLI2 has been shown to bind under similar conditions [33]. These results support and extend a previous report [84] by showing that BRG1 can associate with GLI2-specific *Mef2c* sites *C* and *D*, which are closer to the ISL-1-dependent and NKX2-5/FOXH1-dependent SHF enhancer regions than sites previously identified (Fig. 5b) [84]. Of these two sites, Brg1 is more efficiently recruited to *Mef2c* site *C* in P19 cells overexpressing Gli2. Moreover, when HH signalling in P19 cells is blocked, there is a reduction of Brg1 association with this site. Thus, our findings indicate that BRG1 is recruited to at least one *Mef2c* gene regulatory element in a HH- and GLI2-dependent manner.

Our hypothesis that Gli2 and Brg1 may share common chromatin targets is also supported by the identification of additional 1892 putative GLI- and BRG1-target genes through an *in silico* analysis (Additional file 5: Table S1), where BRG1 genome-wide ChIP-sequencing peaks from [57] were screened for conserved GLI consensus binding motifs. While we did not identify *Mef2c* as a common putative target, most probably due to the stringent conditions of the analysis, we did identify previously reported shared GLI- and BRG1-target genes, *Gli1* and *Ptch1* [42]*,* amongst the theoretical targets, validating this bioinformatic assay (Additional file 5: Table S1).

A gene ontology analysis identified a broad spectrum of biological processes that are significantly enriched for these genes, such as regulation of gene expression, cell differentiation and system development (Table 4). As GLI2 and BRG1 are ubiquitously expressed proteins [57, 95], it is possible that they might co-regulate a variety of cellular and developmental programs. Notably, we identified nervous system development, where GLI2 and BRG1 are known to interact [42], and heart development (Table 4). *Foxh1*, *Tbx20, Notch1,* and *Wnt5a* are amongst the potential targets in the heart development category. *Foxh1* and *Tbx20* are expressed in the SHF field, while Notch and Wnt signalling pathways are known to regulate heart development [5]. Thus, GLI2 and BRG1 may co-regulate cardiac genes that have yet to be identified. Moreover, we predict that this co-regulation may be common to other developmental programs, which is supported by the identification of additional HH-dependent GO categories, such as osteoblast differentiation, nervous system development and cell cycle regulation [66].

**Table 4:** Selected gene ontology biological processes significantly enriched among genes within 50 kb of a BRG1- associating site and GLI consensus binding motif^a^

Category	Targets	Hypergeometric	Example genes
		*P*-value	
Regulation of gene expression	501	3.35E-25	*Atf2, Meis1, Ncoa2, Ncor1, Smad2*
Cellular process	1211	4.15E-25	*Actn1, Ctnna1, Ctnnd1, Mapt, Myo1e*
System development	418	1.04E-22	*Angpt1, Fgf15, Fgf18, Pdgfa*
Cell differentiation	342	2.50E-20	*Creb1, Dhh, Mef2d, Ptch1, Rara, Smo*
Nervous system development	235	6.98E-17	*Bdnf, Neurod4, Nkx2-2, Pax6*
Pattern specification process	77	4.21E-10	*Hoxa2, Hoxa9, Hoxd3, Yy1*
Ear morphogenesis	25	3.67E-09	*Atoh1, Otx1, Otx2, Gata2*
Heart development	69	5.55E-09	*Foxh1, Notch1, Tbx20, Ttn, Wnt5a*
Regulation of cell cycle	99	4.45E-07	*Cdc26, Cdk4, Cdk6, E2f1, E2f2*
Osteoblast differentiation	20	7.13E-06	*Bmp2, Gli1, Gli2*
Cell migration	79	1.05E-05	*Epha2, Fat1, Lama5, Lamc1, Tubb2b*
Chromosome organization	108	4.83E-05	*Hdac3, Mll3, Myst2, Smarca2, Smarcd1*

^a^A complete list can be found in Additional file 5: Table S1

## Conclusion

Taken together, our results indicate that GLI2 and BRG1 co-operate to regulate cardiomyogenesis in vitro. We propose a mechanism where HH signaling, through activated GLI2, recruits BRG1 along with other potential co-factors to the *Mef2c gene* site *C* to promote *Mef2c* expression (Fig. 6b). Inhibition of HH signaling results in reduced BRG1 association at *Mef2c* site *C* and reduced *Mef2c* expression (Fig. 6c). Since BRG1 association correlates with chromatin accessibility [84], we predict that GLI2 might recruit BRG1 to increase chromatin accessibility on *Mef2c* and other potential yet unidentified target genes to regulate in vitro cardiomyogenesis.

## Abbreviations

ChIP, chromatin immunoprecipitation; DHH, desert hedgehog; DMSO, dimethylsulphoxide; EC, embryonal carcinoma; EGS, ethylene glycol bis[succinimidylsuccinate]; EnR, engrailed repression domain; FHF, first heart field; HH, hedgehog; HRP, horseradish peroxidase; IHH, Indian hedgehog; LIF, leukemia inhibitory factor; MEF, mouse embryonic fibroblast; mES, mouse embryonic stem; MyHC, myosin heavy chain; PTA, persistent truncus arteriosus; PVDF, polyvinylidene fluoride; RIPA, radioimmunoprecipitation; SAG, smoothened agonist; SHF, secondary heart field; SHH, sonic hedgehog; SWI/SNF, switch/sucrose non-fermentable; TBST, tris-buffered saline and Tween 20

## Additional files

Additional file 1: Figure S1. mRNA expression levels of the Gli2S662A-Flag transgene in mESC clones stably transfected with a Gli2 expression plasmid or with the empty vector. Total RNA was extracted from cells at various time points during differentiation. Expression values are relative to those at day 0 with the clone “mESC[Flag]#1” and are normalized to *β-actin*. For each clone, *n* = 1. (PDF 367 kb) Additional file 2: Figure S2. GLI2 may regulate early neurogenesis during mES cell differentiation. Total RNA was isolated from differentiating mES[GLI2] and mES[Ctrl] cultures on days indicated and analyzed using qPCR for the expression of (A) *Pax3* and (B) *Ascl1*. Expression levels were normalized to *β-actin*, calibrated to day 0 mES[Ctrl] culture expression levels, and presented as a percentage of the highest expression level recorded, per gene. Error bars represent +/- SEM; *n* = 3. One-tailed Student’s T-tests were used for statistical analyses. Grey lines represent paired T-tests; black lines represent unpaired T-tests; *(star symbol) p* < 0.05. (PDF 340 kb) Additional file 3: Figure S3. Modulation of HH signalling does not affect cell proliferation or survival. (A) Day 7 cells were treated with EdU for 1 h prior to staining. At least 5000 cells per sample, across 20 fields of view, were counted. Error bars represent +/- SEM. Student T-tests were used for statistical analyses. *n* = 3. (B) The yields of total RNA extracted from mES[Ctrl] and mES[GLI2] are given as a proxy for cell numbers in the cultures. For each time point, n is between 3 and 6, and error bars represent +/- SEM. (C) The proportion of cells with highly condensed, apoptotic nuclei after Hoechst staining at day 7 is given. At least 2500 cells per condition per replicate were counted. *n* = 2. (D) Yields of RNA in mES cultures treated with methanol vehicle or KAAD-cyclopamine. For each time point, *n* = 3, and error bars represent +/- SEM. (E) The proportion of cells with highly condensed, apoptotic nuclei is given for vehicle- and KAAD-cyclopamine-treated mES cultures at day 7. *n* = 2, and at least 500 cells per replicate and condition were counted. (PDF 415 kb) Additional file 4: Figure S4. Expression of Brg1 in mES and P19 cells overexpressing Gli2. qPCR analysis of *Brg1* mRNA expression levels in (A) mES[Ctrl] (white bars) and mES[GLI2] (grey bars) cells, or (B) P19[Ctrl] (white bars) and P19[GLI2] (grey bars) cells. For (A-B) expression levels were normalized to *β-actin*, calibrated to day 0 mES[Ctrl] or P19[Ctrl] culture expression levels, and presented as a percentage of the highest expression level recorded, per gene. Two-tailed Student’s T-tests were used for the mRNA statistical analyses; *n* = 3. (PDF 341 kb) Additional file 5: Table S1. A complete list of gene ontology biological processes significantly enriched among genes within 50 kb of a BRG1-associating site and GLI consensus binding motif. (XLSX 490 kb)
