# Supplementary figures and images for: BRG1 interacts with GLI2 and binds Mef2c gene in a hedgehog signalling dependent manner during in vitro cardiomyogenesis
Source: BMC Dev Biol. 2016 Aug 2;16:27. doi: 10.1186/s12861-016-0127-8 (PMC4970297; doi:10.1186/s12861-016-0127-8)

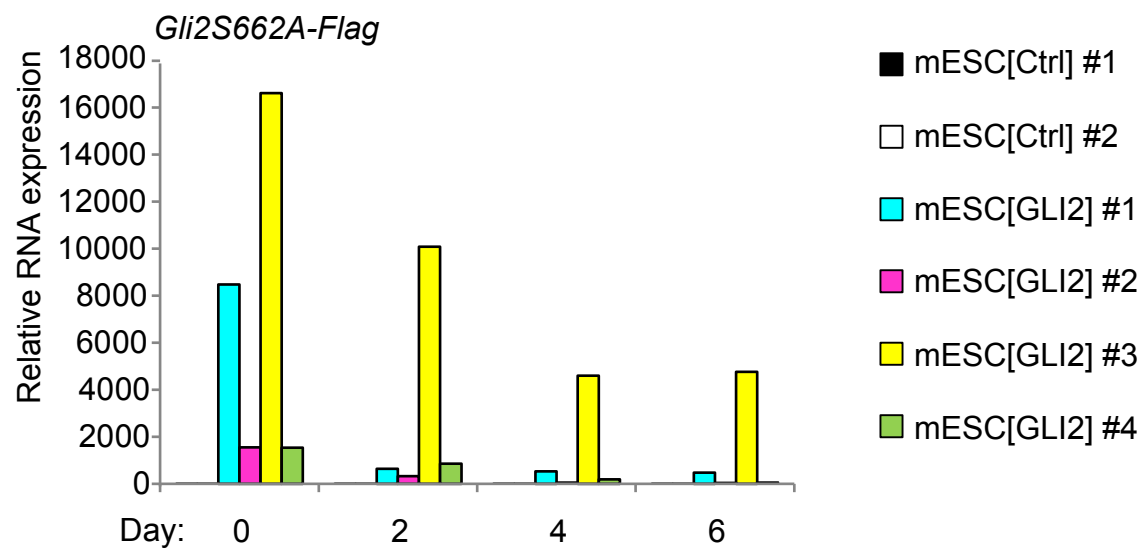

Supplement: Additional file 1: Figure S1. — mRNA expression levels of the Gli2S662A-Flag transgene in mESC clones stably transfected with a Gli2 expression plasmid or with the empty vector. Total RNA was extracted from cells at various time points during differentiation. Expression values are relative to those at day 0 with the clone “mESC[Flag]#1” and are normalized to β-actin. For each clone, n = 1. (PDF 367 kb) [file 12861_2016_127_MOESM1_ESM.pdf]

**A**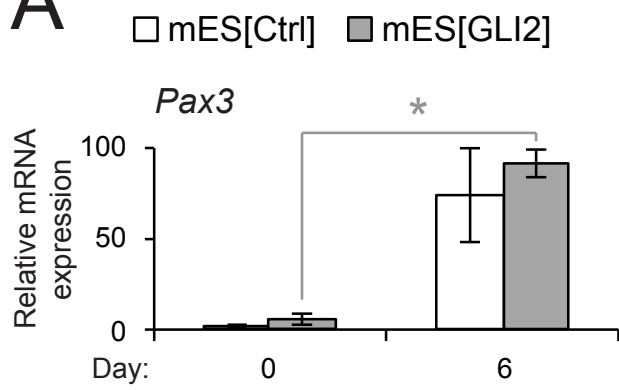**B**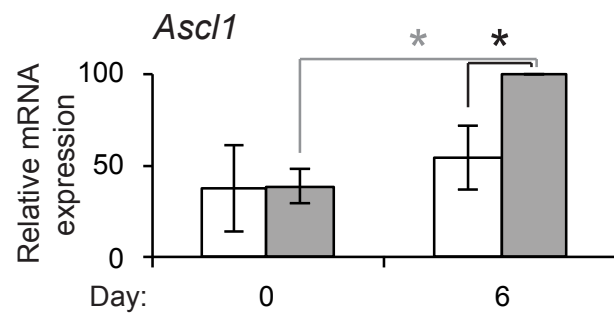

Supplement: Additional file 2: Figure S2. — GLI2 may regulate early neurogenesis during mES cell differentiation. Total RNA was isolated from differentiating mES[GLI2] and mES[Ctrl] cultures on days indicated and analyzed using qPCR for the expression of (A) Pax3 and (B) Ascl1. Expression levels were normalized to β-actin, calibrated to day 0 mES[Ctrl] culture expression levels, and presented as a percentage of the highest expression level recorded, per gene. Error bars represent +/- SEM; n = 3. One-tailed Student’s T-tests were used for statistical analyses. Grey lines represent paired T-tests; black lines represent unpaired T-tests; (star symbol) p < 0.05. (PDF 340 kb) [file 12861_2016_127_MOESM2_ESM.pdf]

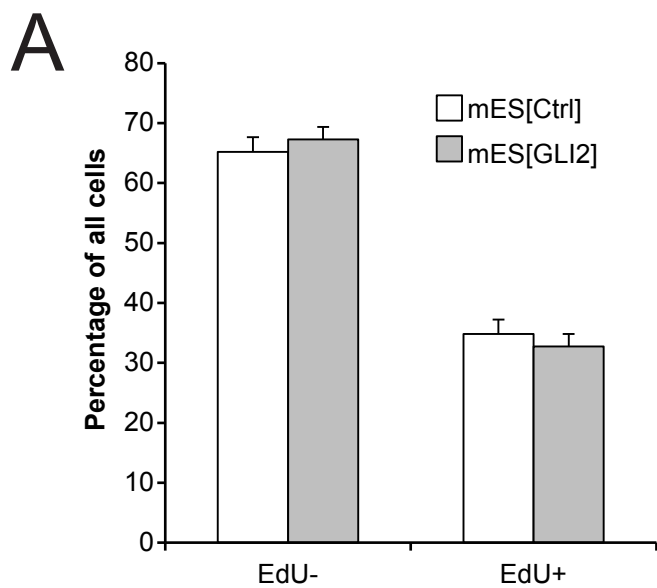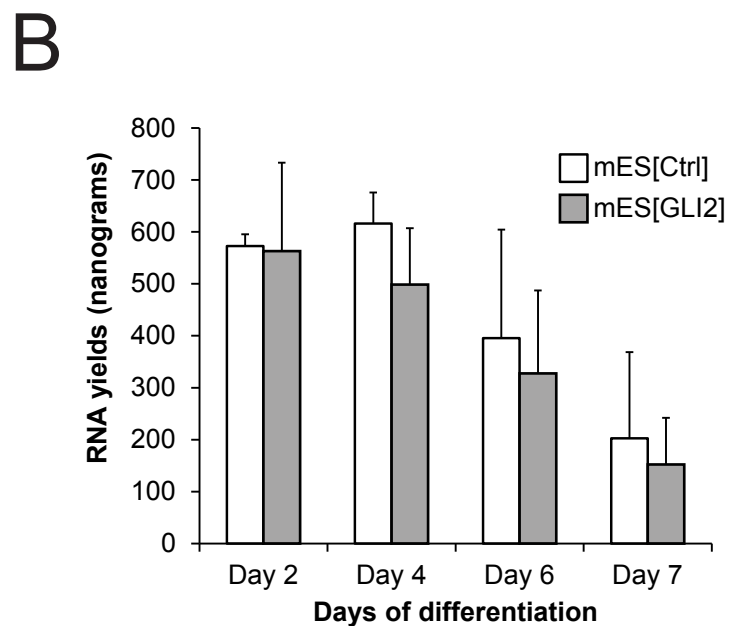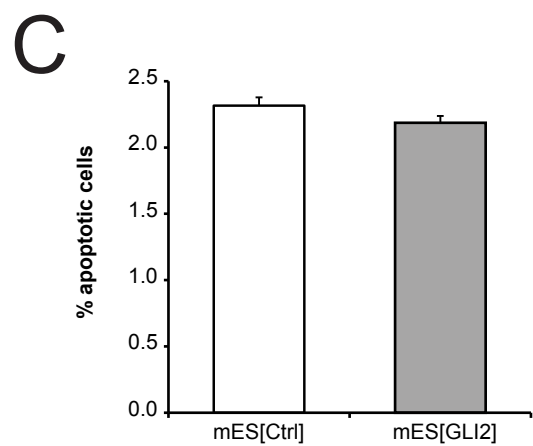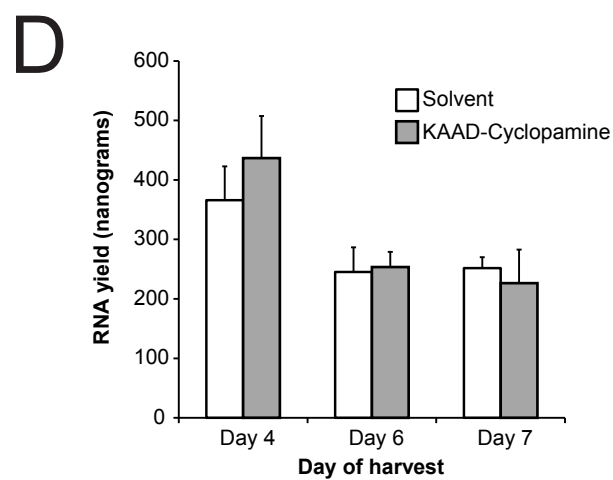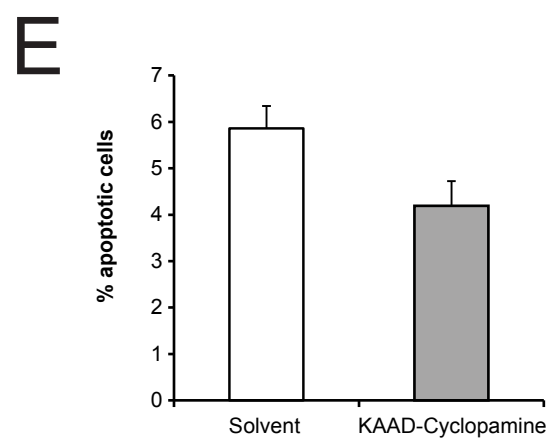

Supplement: Additional file 3: Figure S3. — Modulation of HH signalling does not affect cell proliferation or survival. (A) Day 7 cells were treated with EdU for 1 h prior to staining. At least 5000 cells per sample, across 20 fields of view, were counted. Error bars represent +/- SEM. Student T-tests were used for statistical analyses. n = 3. (B) The yields of total RNA extracted from mES[Ctrl] and mES[GLI2] are given as a proxy for cell numbers in the cultures. For each time point, n is between 3 and 6, and error bars represent +/- SEM. (C) The proportion of cells with highly condensed, apoptotic nuclei after Hoechst staining at day 7 is given. At least 2500 cells per condition per replicate were counted. n = 2. (D) Yields of RNA in mES cultures treated with methanol vehicle or KAAD-cyclopamine. For each time point, n = 3, and error bars represent +/- SEM. (E) The proportion of cells with highly condensed, apoptotic nuclei is given for vehicle- and KAAD-cyclopamine-treated mES cultures at day 7. n = 2, and at least 500 cells per replicate and condition were counted. (PDF 415 kb) [file 12861_2016_127_MOESM3_ESM.pdf]

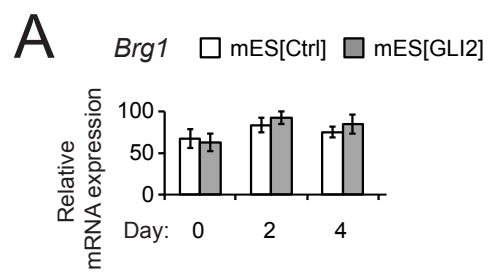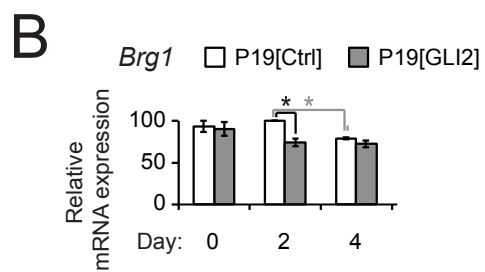

Supplement: Additional file 4: Figure S4. — Expression of Brg1 in mES and P19 cells overexpressing Gli2. qPCR analysis of Brg1 mRNA expression levels in (A) mES[Ctrl] (white bars) and mES[GLI2] (grey bars) cells, or (B) P19[Ctrl] (white bars) and P19[GLI2] (grey bars) cells. For (A-B) expression levels were normalized to β-actin, calibrated to day 0 mES[Ctrl] or P19[Ctrl] culture expression levels, and presented as a percentage of the highest expression level recorded, per gene. Two-tailed Student’s T-tests were used for the mRNA statistical analyses; n = 3. (PDF 341 kb) [file 12861_2016_127_MOESM4_ESM.pdf]
